# Knowledge Domain and Emerging Trends in Podocyte Injury Research From 1994 to 2021: A Bibliometric and Visualized Analysis

**DOI:** 10.3389/fphar.2021.772386

**Published:** 2021-12-03

**Authors:** Tongtong Liu, Liping Yang, Huimin Mao, Fang Ma, Yuyang Wang, Yongli Zhan

**Affiliations:** Guang’anmen Hospital, China Academy of Chinese Medical Sciences, Beijing, China

**Keywords:** bibliometrics analysis, visualized, podocyte injury, citespace, VOSviewer, histcite, bibliometrix

## Abstract

**Background:** Podocyte injury has a direct causal relationship with proteinuria and glomerulosclerosis and, on a chronic level, can lead to irreversible disease progression. Podocyte injury plays a critically decisive role in the development of proteinuric kidney disease. In recent years, the research on podocyte injury has developed rapidly all over the world. However, no report has summarized the field of podocyte injury as a whole to date. Using bibliometric analysis, this study aimed to evaluate the current state of worldwide podocyte injury research in the last 30 years and identify important achievements, primary research fields, and emerging trends.

**Methods:** Publications related to podocyte injury were retrieved from Web of Science Core Collection. HistCite, VOSviewer, CiteSpace, and the Bibliometrix Package were used for bibliometric analysis and visualization, including the analysis of the overall distribution of annual outputs, leading countries, active institutions and authors, core journals, co-cited references, and keywords. Total global citation score and total local citation score were used to assess the quality and impact of publications.

**Results:** A total of 2,669 publications related to podocyte injury were identified. Publications related to podocyte injury tended to increase continuously. A total of 10,328 authors from 2,171 institutions in 69 countries published studies related to podocyte injury. China (39.46%) was the most prolific country, and the number of citations of studies in the United States (cited 36,896 times) ranked first. Moin A Saleem, John Cijiang He, and Zhihong Liu were the top three contributing authors, and Journal of the American Society of Nephrology and Kidney International were the most popular journals in the field. “Diabetic nephropathy” is the primary focus area of podocyte injury research, and “autophagy,” “microRNA,” and “inflammation” were the top keywords of emerging research hotspots, and traditional Chinese medicine monomer may be a neglected research gap.

**Conclusion:** Our research found that global publications on podocyte injury have increased dramatically. Diabetic nephropathy is the main research field of podocyte injury, whereas autophagy, microRNA, and inflammation are the top topics getting current attention from scholars and which may become the next focus in podocyte injury research.

## Introduction

Podocytes are highly differentiated epithelial cells attached to the surface of the glomerular basement membrane. They play a prominent role in maintaining the integrity of the glomerular filtration barrier. Podocyte injury and loss are closely related to the development of proteinuria and glomerulosclerosis. In 1994, Kretzler M et al. first attributed glomerulosclerosis to podocyte depletion in a rat model (Kretzler et al., 1994). An important study conducted by Pagtalunan ME et al. found that podocyte loss was related to albuminuria and the deterioration of renal function in Pima Indians with type II diabetes (Pagtalunan et al., 1997). Podocyte depletion is an important morphologic characteristic in the pathogenesis of diabetic kidney disease (DKD) and focal segmental glomerulonephritis (FSGS). Many studies have shown that podocyte injury occur in early-stage DKD (Coimbra et al., 2000) and FSGS (Alachkar et al., 2013), and uncontrollable persistent podocyte injury will lead to glomerulosclerosis and disease progression (Wharram et al., 2005; Makino et al., 2021). Therefore, research focused on preventing podocyte injury or promoting podocyte repair has been the focus and hotspot in this field.

Podocyte injury manifests as hypertrophy, foot process effacement, autophagy, mesenchymal transition, detachment, and apoptosis (Zhou L. et al., 2019). Stress factors that mediate the injury process include mechanical stress (glomerular hypertension or hyperfiltration), oxidative stress, and immune (inflammatory) stress (Nagata, 2016). Once the podocytes are injured, the structure of actin cytoskeleton changes supervenes such that damage to the renal filtration barrier progresses until proteinuria, and glomerular disease occurs (Schell and Huber, 2017). On the other hand, podocyte injury inhibits the synthesis of vascular endothelial growth factor, which in turn affects vascular compartment stabilization and further interrupts the crosstalk between podocytes and vascular compartments, and ultimately leading to the development of glomerulosclerosis. Notably, the proliferation of podocytes is very limited. Although studies have found that podocytes have a certain reserve capacity in infancy, this reserve is rather small (<10% of all podocytes), and becomes depleted with age (Berger et al., 2014; Eng et al., 2015). Mild podocyte injury may be reversible, however, until a certain threshold in podocyte loss is reached (>40% podocyte depletion), and inevitable renal failure occurs (Wharram et al., 2005). Hence, research on podocyte injury is a very promising field, and conducting an analysis of the current status, focus areas, and emerging trends in the field of podocyte injury will yield significant findings.

Bibliometrics is a convenient new approach to the qualitative and quantitative analyses of publications (Chen, 2004; 2017). Using this method, researchers can quickly dig deep into the thematic evolution, main research fields, and new research directions in a certain research field (Chen and Song, 2019). Bibliometrics has become widely used in many disciplines as an auxiliary research method (Wang et al., 2021). However, bibliometric studies on podocyte injury remains lacking.

In this study, we used bibliometric methods to analyze the publications on podocyte injury and systematically evaluate the research status, current research focus, emerging research trends of podocyte injury in the past three decades, highlighting landmark achievements, and pointing out directions for future research.

## Materials and Methods

### Data Source and Search Strategy

We conducted a literature search on the Web of Science Core Collection (WoSCC) on podocyte injury in the past 30 years (from 1990 to 2021). The search formula was as follows: TS = (“podocyte pyroptosis” OR “podocyte apoptosis” OR “podocytopathy” OR “podocytopathies” OR “podocyte injury” OR “podocyte damage”, and OR “podocyte dysfunction”). The article language was set to English. In order to avoid deviations from data updates, all the above operations were performed within 1 day, and on July 4, 2021.

### Eligibility Criteria and Data Collection

The document types included in the study were only articles and reviews. Meeting abstracts, editorial materials, and proceedings papers, among others, and were excluded. Duplicate studies were also removed artificially. All the information, including the number of papers and citations, titles, authors, affiliations, countries, keywords, journal, publication year, and references, were collected for bibliometric analysis.

### Statistical Analysis

In this study, HistCite (version 12.03.17), VOSviewer (version 1.6.16), CiteSpace (version 5.7.R5), and the Bibliometrix 4.1.0 Package (https://www.bibliometrix.org) based on the R language were used to perform the bibliometric analysis.

HistCite (Garfield et al., 2006) was used to calculate the total number of publication records, total global citation score (TGCS), and total local citation score (TLCS) for each publication year, active countries, top institutions, core journals, and authors. More importantly, it was used to identify the representative citation paths of important references.

VOSviewer (van Eck and Waltman, 2010) was used to visualize complex co-citation networks, such as the cooperation and time trends among countries, institutions, and individuals. The size of the nodes represents the number of publications; the thickness of the line represents the strength of the link; and the colors of the nodes represent different clusters or times.

CiteSpace was used to aid visual analysis of the knowledge domain and emerging trends (Chen, 2004), including cluster analysis, dual-map overlay of citations, timeline or time zone views, references, and keywords citation bursts (Chen and Leydesdorff, 2014; Chen and Song, 2019). Cluster analysis can classify references and keywords and identify important research areas on podocyte injury. The modularity Q and mean silhouette are two important evaluation indicators in cluster analysis. Q > 0.3 indicates that the clustering structure is significant enough. Mean silhouette >0.5 indicates that the clustering results are convincing. The bursts of keywords and references are often used to detect new research trends in the field.

The Bibliometrix Package is an established tool based on the R language that is used for bibliometric analysis (Aria and Cuccurullo, 2017). We conducted a thematic evolution analysis using the Bibliometrix Package to categorize the changes in podocyte injury research into different periods.

## Results

### Overall Distribution

**FIGURE 1:**
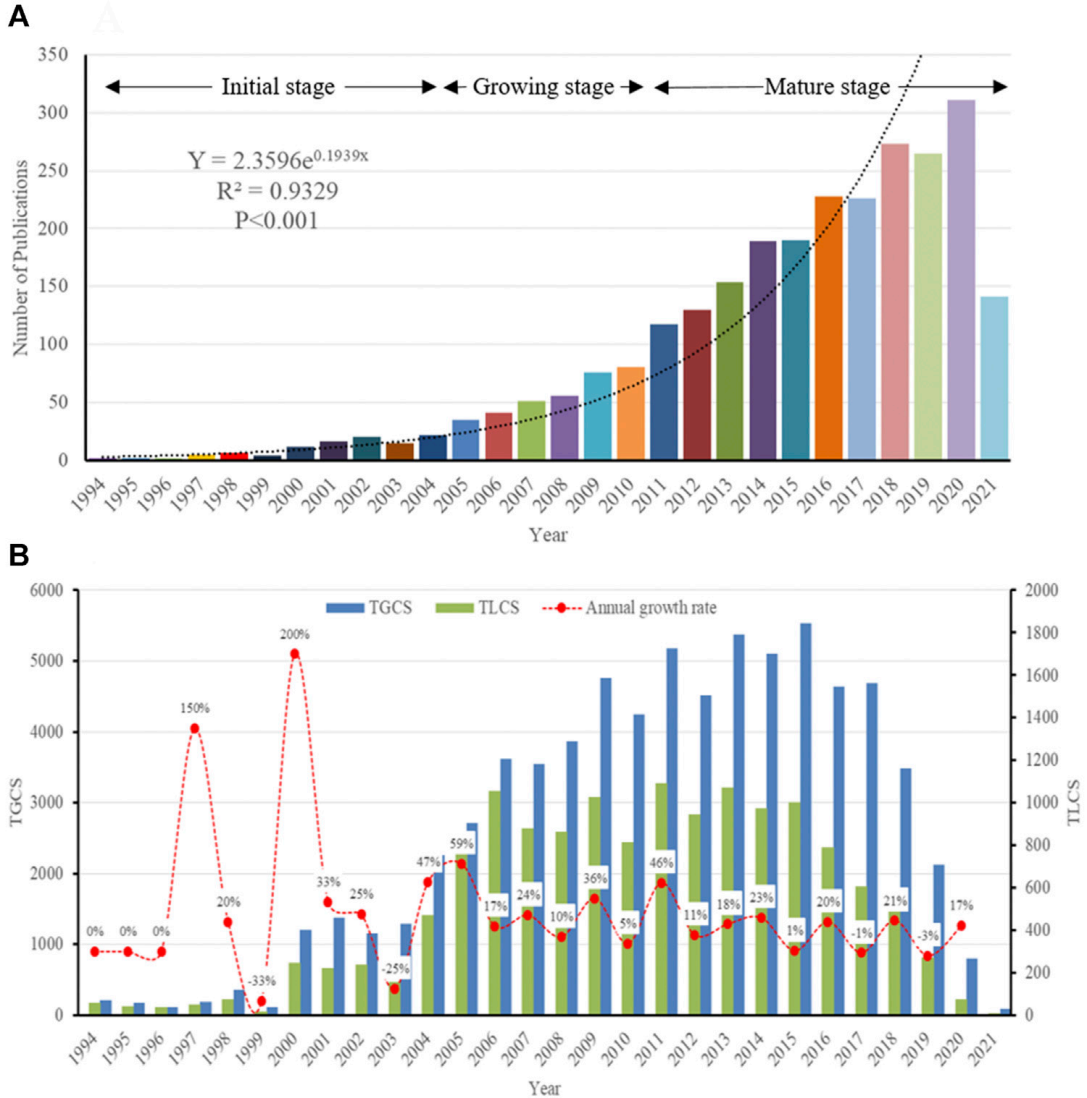
Overall Distribution of publication outputs on podocyte injury **(A)** Global annual output trends; **(B)** TGCS, TLCS and growth rate in annual publications.

A total of 2,669 publications related to podocyte injury were retrieved from WoSCC, including 2,311 articles, and 358 reviews (Supplementary Table S1; Supplementary Figure S1). Curve fitting analysis showed that the annual number of publications on podocyte injury has undergone an overall increasing trend since 1994 (R^2^ = 0.9329; *p* < 0.001). We artificially divide this period into three stages according to the annual output and growth rate: Initial stage (1994–2004), growing stage (2005–2010), and mature stage (2011–2021). In the initial stage, the total number of publications on podocyte injury was less than 20 per year, and in 1994–1996, only two articles were published in each year, and the only two articles in 1994 were written by Pascua M and Kretzler M, who investigated podocyte injury earlier than researchers in other parts of the world. Pascual et al. (1994) found that complement receptor 1 (CR1) can be used as a marker for podocyte injury (cited 68 times). Over the same period, Kretzler et al. (1994) proposed for the first time that in the uninephrectomized-desoxycorticosterone hypertensive rat model, and glomerulosclerosis may be due to podocyte injury (cited 143 times). In the growing stage, the number of total publications on podocyte injury was less than 100 per year, but increased at an average rate of 9.6 articles per year, and with an average annual growth rate of 25.23%. In the mature stage, the total number of publications on podocyte injury was more than 100 per year and increased at an average rate of 23.1 articles per year, with an average annual growth rate of 14.76% (Figure 1A). The highest number of articles was published in 2020 (*n* = 311), which was more than the total number of articles published in the previous 15 years (1994–2008, *n* = 289).

So far, these articles have been cited 72,836 times, with an average of 27.29 times per article. Since the field of research was then still immature, the TGCS and TLCS of articles published in the initial stage were low. However, the TGCS increased year by year from 2005 to 2008. Since 2009, the TGCS has been relatively stable, indicating that the research on podocyte injury has entered a relatively mature stage (Figure 1B).

### Leading Countries

**FIGURE 2:**
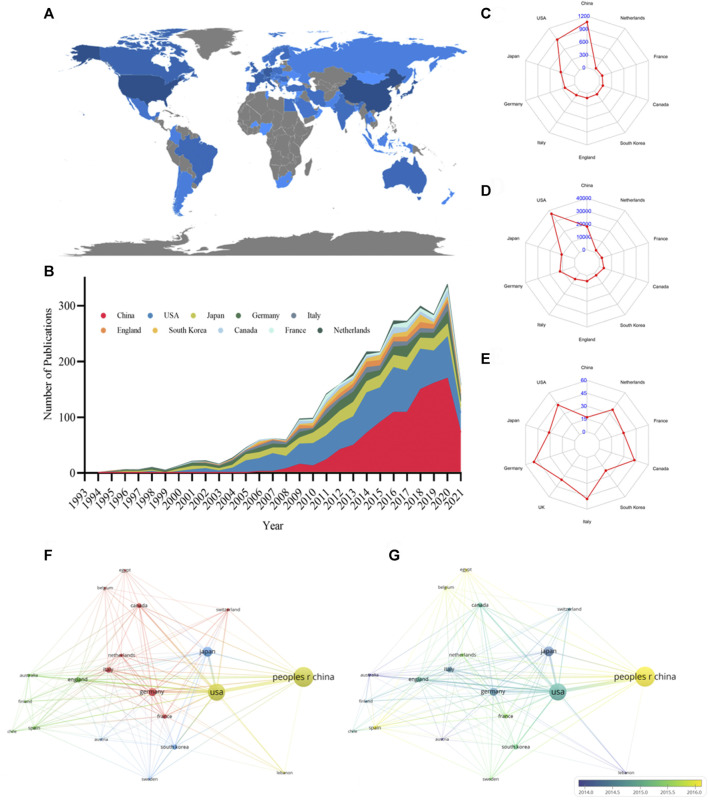
Leading countries in podocyte injury research. **(A)** Geographical distribution of global output; **(B)** Annual output trend of the top 10 productive countries; **(C)** Radar map of the top 10 productive countries; **(D)** Radar map of TGCS of the top 10 productive countries; **(E)** Radar map of average cited of the top 10 productive countries; **(F)** Visual cluster analysis of cooperation among countries; **(G)** Timeline visualization of cooperation among countries.

From 1994 to 2021, 69 countries published research articles on podocyte injury. The global article productivity is presented in Figure 2A. The top 10 countries with the highest number of publications have generated about 84.57% of the articles in the world (Figure 2B; Table 1). China showed the highest output, publishing a total of 1,065 (39.46%) articles related to podocyte injury, followed by the United States (*n* = 872; 32.31%), and Japan (*n* = 341; 12.63%) (Figure 2C). The most cited country for published articles is the United States (cited 36,896 times), followed by China (cited 18,077 times), and Germany (cited 12,008 times) (Figure 2D). In addition, German publications have the highest average number of citations (average cited 49.83 times), followed by Italy (average cited 48.02 times), and Canada (average cited 42.79 times) (Figure 2E). The visualized international collaboration network showed that the cooperation between countries is relatively close. The United States and China showed the closest cooperation, and the United States has been in cooperation with almost all the other countries (Figure 2F). Since 1994, research in this field has increased in Germany, Italy, and the United Kingdom, whereas the research on podocyte injury in China, Spain, and Egypt has increased since 2005 (Figure 2G).

**TABLE 1:** The top 10 productive countries concerning podocyte injury research.

Rank	Country	Publications n (%)	LCS	TGCS	Average citation	Centrality
1	China	1,065 (39.90%)	3,739	18,077	16.97	0.03
2	United States	878 (32.67%)	7,651	36,896	42.31	0.54
3	Japan	342 (12.78%)	2,453	10,689	31.35	0.08
4	Germany	242 (9.03%)	2,399	12,008	49.83	0.29
5	Italy	120 (4.57%)	1,030	5,714	35.28	0.16
6	England	107 (4.46%)	648	4,304	48.02	0.09
7	South Korea	93 (3.48%)	393	2056	22.11	0.03
8	Canada	86 (3.22%)	627	3,680	42.79	0.06
9	France	72 (2.66%)	338	2,100	29.58	0.16
10	Netherlands	54 (2.02%)	425	1919	35.54	0.06

### Active Institutes and Authors

**FIGURE 3:**
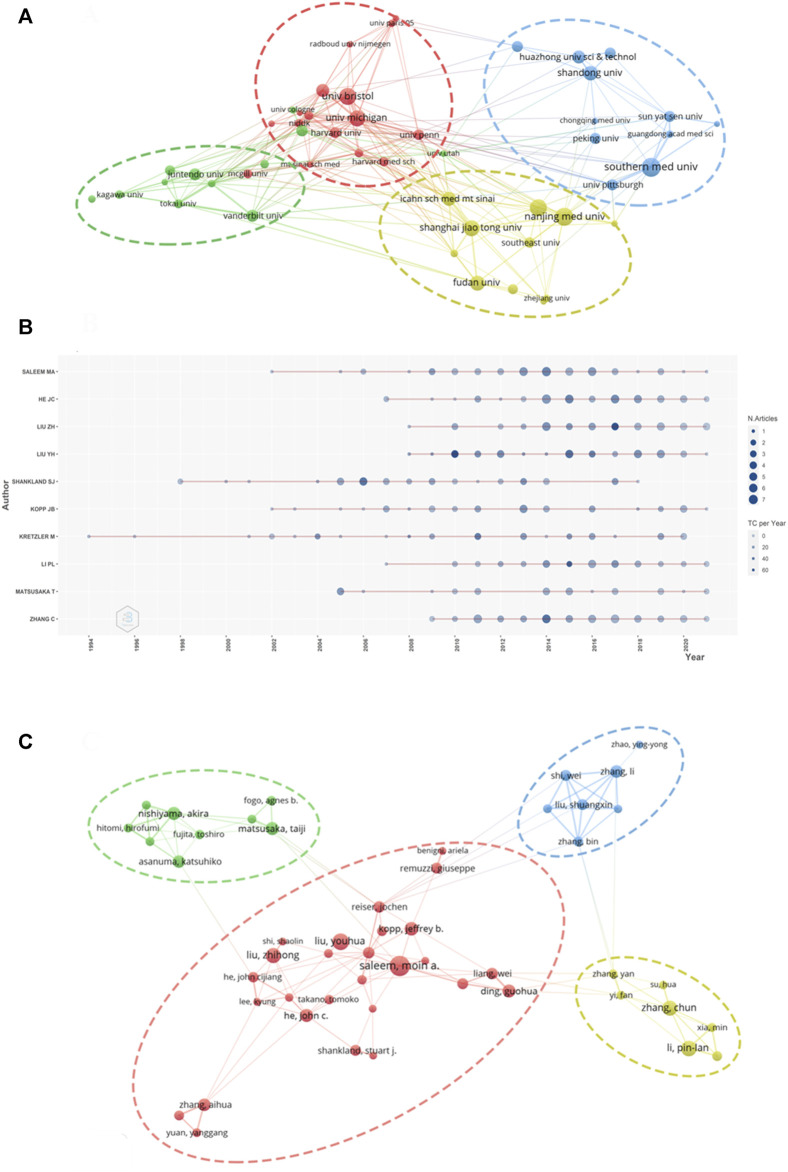
Visualization of active institutes and authors analysis **(A)** Cluster analysis of cooperation among institutes; **(B)** Timeline distribution of the top 10 most productive authors; **(C)** Cluster analysis of cooperation among authors.

A total of 10,328 authors from 2,172 institutions have published articles on podocyte injury. The top 10 institutions with the highest output in podocyte injury research are shown in Table 2. Southern Medical University in China (*n* = 68) was the leading institution in terms of output, followed by Nanjing Medical University in China (*n* = 64), the University of Bristol in the United Kingdom (n = 62), and the University of Michigan in the United States (*n* = 62). The TGCS of the University of Washington in the United States (cited 3,986 times) was the highest, followed by the University of Michigan (cited 3,280 times), and the University of Bristol (cited 2067 times). Cooperation among institutions was relatively close and was divided into four institutional clusters. The cooperation groups led by the University of Michigan showed the closest cooperation with other institutions (Figure 3A).

**TABLE 2:** The top 10 productive institutions concerning podocyte injury research.

Institution	Country	Publication counts	TGCS	TLCS	Average citation
Southern Medical University	China	68	1951	371	28.69
Nanjing Medical University	China	64	1,426	373	22.28
University of Bristol	United Kingdom	62	2067	328	33.34
University of Michigan	United States	62	3,280	739	52.90
Nanjing University	China	60	1,645	352	27.42
University of Washington	United States	57	3,986	1,051	69.93
Shanghai Jiao Tong University	China	56	1,177	193	21.02
Fudan University	China	53	903	191	17.04
Shandong University	China	49	903	197	18.43
Icahn School of Medicine at Mount Sinai	United States	47	973	230	20.70

The top three most productive authors were Moin A Saleem of the University of Bristol (published 50 articles), John Cijiang He of the Icahn School of Medicine at Mount Sinai (published 43 articles), and Zhihong Liu of the Medical School of Nanjing University (published 40 articles) (Table 3). Kretzler M is an early researcher on podocyte injury. He published his first achievement in this field in 1994. So far, he has been engaged in research in this field for nearly 30 years (1994–2020) (Figure 3B). Youhua Liu of Southern Medical University was the most cited author (cited 3,120 times), followed by Stuart J Shankland of the University of Washington (cited 2,730 times) and Matthias Kretzler of the University of Michigan (cited 2,613 times). The degree of cooperation among authors was relatively low and characterized by cooperation within the institution (Figure 3C).

**TABLE 3:** The top 10 most productive authors in podocyte injury research.

Rank	Name	Country	Institution	Counts	TLCS	TGCS	H-index
1	Moin A Saleem	United Kingdom	Univ Bristol	50	280	1792	24
2	John Cijiang He	United States	Icahn Sch Med Mt Sinai	43	309	1,212	20
3	Zhihong Liu	China	Nanjing Med Univ	40	317	1,365	18
4	Youhua Liu	China	Southern Med Univ	35	688	3,120	25
5	Stuart J Shankland	United States	Univ Washington	37	872	2,730	29
6	Jeffrey B Kopp	United States	NIH	35	270	1,417	22
7	Pin-Lan Li	United States	Virginia Commonwealth Univ	32	279	1,330	19
8	Matthias Kretzler	United States	Univ Michigan	32	563	2,613	24
9	Taiji Matsusaka	Japan	Tokai Univ	29	282	927	15
10	Chun Zhang	China	Huazhong Univ Sci & Technol	29	286	1,004	16

### Core Journals

**FIGURE 4:**
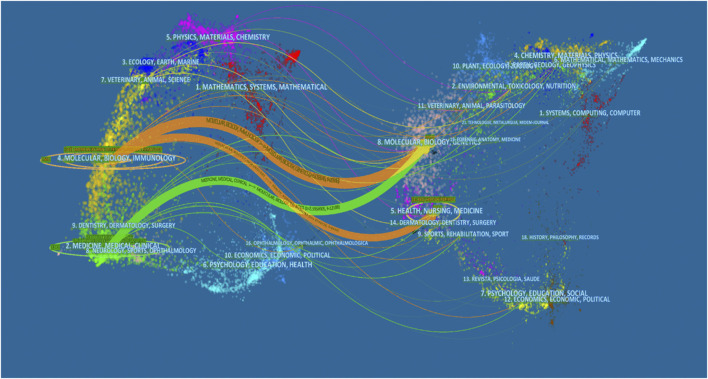
The dual-map overlay of articles citing on podocyte injury research. (The left side were the citing journal, the right side were the cited journal, and the line path represents the citation relationship).

All articles on podocyte injury were published in 413 journals. The top 10 journals with the highest productivity are shown in Table 4. About 31.32% of the articles were published in these journals. The Journal of the American Society of Nephrology (JASN) (published 159 articles, cited 10,305 times) was the most prolific journal, followed by Kidney International (KI) (published 148 articles, cited 8,895 times) and American Journal of Physiology–Renal Physiology (published 142 articles, cited 4,476 times). These three journals were also the most cited. The dual-map overlay shows three main citation paths. The published articles were mainly focused on journals in the field of molecular, biology, immunology and medicine, and medical clinical, whereas most of the cited articles were published in journals in the field of molecular, biology, genencs, health, nursing, and medicine (Figure 4).

**TABLE 4:** The top 10 core journals on podocyte injury research.

Rank	Journal	Counts	TLCS	TGCS	IF (2020)	H-index
1	Journal of the American Society of Nephrology	159	2,110	10,305	10.121	60
2	Kidney International	148	2072	8,895	10.612	55
3	American Journal of Physiology-Renal Physiology	142	1,027	4,476	3.377	38
4	Plos One	94	0	2,182	3.240	26
5	Nephrology Dialysis Transplantation	75	525	2,440	5.992	30
6	Scientific Reports	67	0	944	4.379	18
7	Biochemical and Biophysical Research Communications	49	176	560	3.575	15
8	American Journal of Pathology	36	553	1752	4.307	24
9	Journal of Biological Chemistry	35	462	1857	5.157	24
10	Molecular Medicine Reports	31	62	291	2.952	10

### Co-Cited References

**FIGURE 5:**
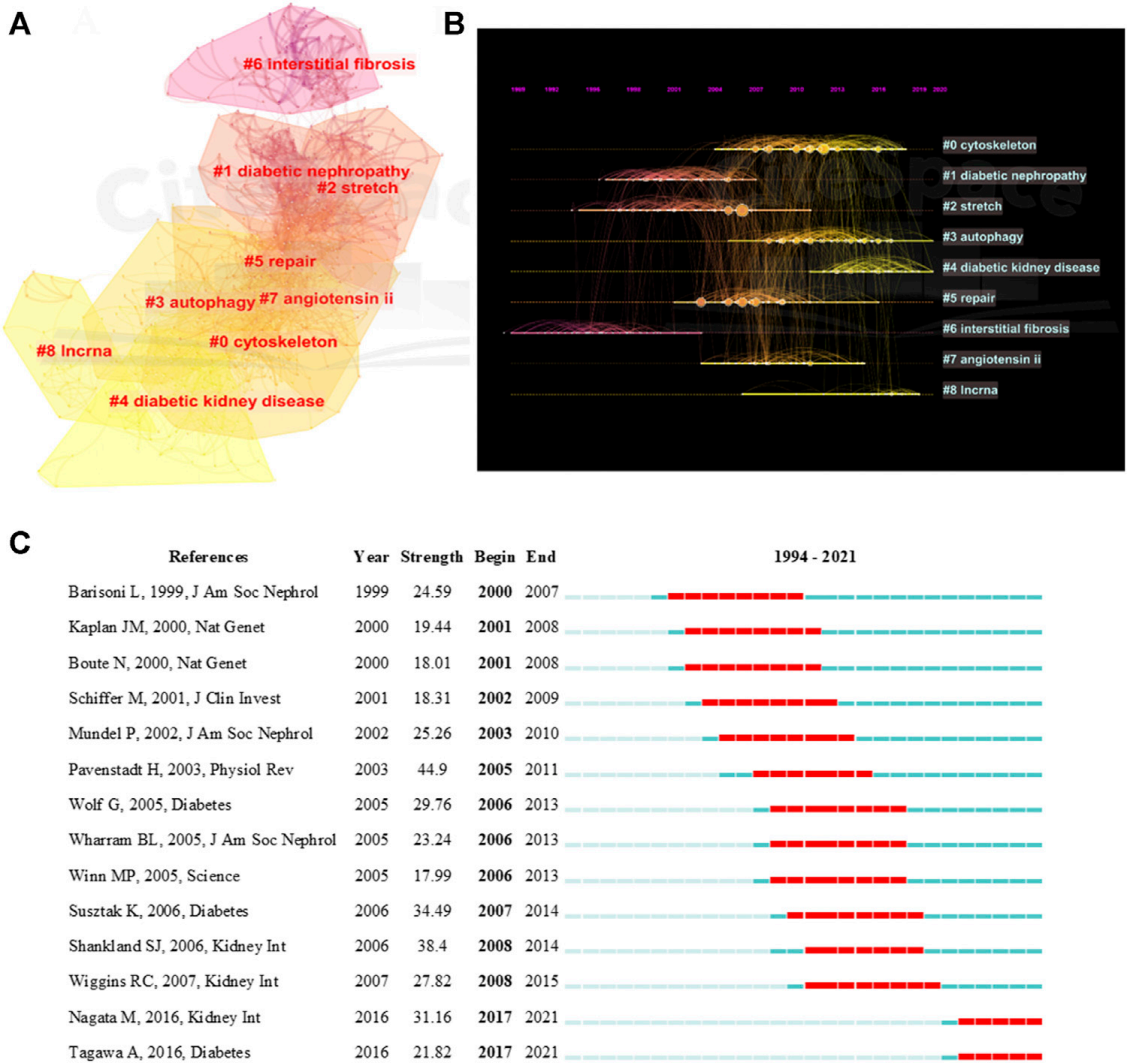
Visualization of co-cited reference analysis. **(A)** Cluster Analysis of Co-cited References; **(B)** Timeline distribution of the top 9 clusters; **(C)** Representative burst references among top 25 references with the strongest citation bursts.

The top 15 most cited references includes 7 reviews and 8 research articles (Table 5). Most articles focused on the pathophysiology and mechanism of podocyte injury. The most cited article was written by Susztak, who reported that a high-glucose environment can cause podocyte depletion, and leading to early DKD (cited 767 times) (Susztak et al., 2006). Subsequently, Inoki K et al. found that reducing the activity of mammalian target of rapamycin complex 1 in podocytes is a potential strategy for preventing DKD (cited 355 times) (Inoki et al., 2011), which further promoted the development of this field of study. We subsequently constructed a visualization network of cited references and performed a cluster analysis. A total of 14 clusters were found, the modularity Q was 0.6587, and the mean silhouette value was 0.8617 (Supplementary Table S2). Six clusters with the highest K values were identified (Table 6; Figure 5A), which include “cytoskeleton,” “diabetic nephropathy,” and “stretch,” among others. Furthermore, we performed a visualized timeline for clusters (Figure 5B). We found that “interstitial fibrosis” is an early field in podocyte injury. However, the current hotspots of podocyte injury are on “autophagy,” “diabetic kidney disease,” and “lncRNA.” Finally, we conducted a reference burst. The top 25 references with the strongest citation bursts are shown in Supplementary Figure S2. Figure 5C shows the most representative references in terms of burst strength, burst duration, and burst time. We found that the works of Pavenstädt et al. (2003) have the highest bursts strength. In his article, he described the relationship between podocyte injury and glomerulosclerosis Pavenstädt et al. (2003), indicating that this is the current focus in podocyte injury research. In addition, Nagata M’s article has had a strong citation burst in recent years. His article introduced the correlation between autophagy and podocyte injury, indicating that this has garnered increased research interest in recent years (Nagata, 2016).

**TABLE 5:** The top 10 literatures with the highest number of citations.

Rank	First author	Journal	Year	IF (2020)	Category	Cluster	TGCS	TLCS
1	Susztak K	Diabetes	2006	9.461	Pathophysiology	#2	767	268
2	Shankland SJ	Kidney International	2006	10.612	Pathophysiology	#5	589	249
3	Liu YH	Journal of the American Society of Nephrology	2010	10.121	Pathophysiology	#3	580	53
4	Wiggins RC	Kidney International	2007	10.612	Pathophysiology	#5	494	191
5	Eddy AA	Lancet	2003	79.321	Pathophysiology	#5	460	19
6	Wolf G	Diabetes	2005	9.461	Treatment	#2	454	185
7	Sharma K	Journal of Clinical Investigation	2008	14.808	Treatment	#3	446	44
8	Reiser J	Journal of Clinical Investigation	2004	14.808	Injury mechanism	#5	384	96
9	Wei C	Nature Medicine	2008	53.44	Injury mechanism	#0	375	82
10	Ronconi E	Journal of the American Society of Nephrology	2009	10.121	Treatment	#5	358	55
11	Inoki K	Journal of Clinical Investigation	2011	14.808	Injury mechanism	#1	355	87
12	Abais JM	Antioxidants & Redox Signaling	2015	8.401	Injury mechanism	#5	347	8
13	Coimbra TM	Kidney International	2000	10.612	Injury mechanism	#4	301	50
14	Trimarchi H	Kidney International	2017	10.612	other	#8	289	4
15	Coughlan MT	Journal of The American Society of Nephrology	2009	10.121	Injury mechanism	#4	287	3

**TABLE 6:** The top 6 clusters of co-cited references with the highest K value in podocyte injury research.

Cluster ID	Size	Silhouette	Mean year (Range)	Top term	Log (likelihood ratio)
#0	92	0.827	2011 (2004–2018)	Cytoskeleton	20.42
#1	86	0.873	2001 (1996–2007)	Diabetic nephropathy	11.38
#2	79	0.841	2001 (1994–2011)	Stretch	14.03
#3	77	0.847	2012 (2005–2020)	Autophagy	59.85
#4	74	0.866	2016 (2011–2020)	Diabetic kidney disease	20.58
#5	68	0.791	2007 (2001–2016)	Repair	15.70

### Analysis of Keywords

**FIGURE 6:**
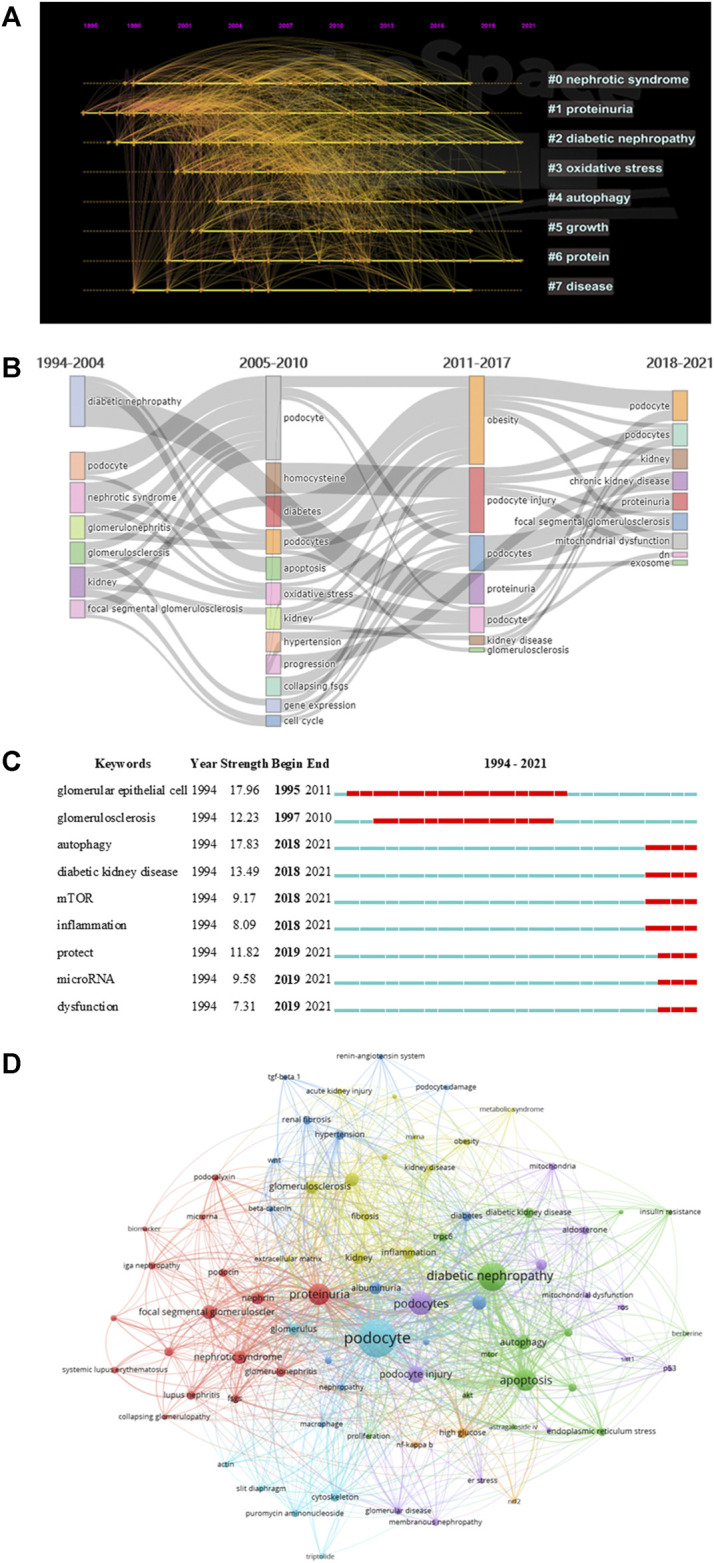
Visualization of keyword analysis. **(A)** Timeline distribution of cluster analysis of keyword; **(B)** Sankey diagram of the keywords evolution of podocyte injury research; **(C)** Representative burst keywords among top 25 references with the strongest citation bursts. **(D)** The network map of keywords.

We extracted 4,080 keywords at data collection, and a cluster analysis revealed 8 clustering results (Table 7). The modularity Q of the cluster was 0.3087, and the mean silhouette value was 0.7322. The timeline of clustering showed that “diabetic nephropathy” and “proteinuria” were the most important areas of podocyte injury research, whereas “autophagy” was an emerging hotspot in podocyte injury studies (Figure 6A). We conducted a thematic evolution analysis on keywords, and found that the initial stages of research on podocyte injury was mainly focused on “diabetic nephropathy.” However, with the maturity of the research field, the main research hotspot of podocyte injury has gradually evolved toward “homocysteine,” “apoptosis,” and “obesity,” among others. In the past 3 years, “mitochondrial dysfunctions,” “exosome,” among others, and has gradually attracted the attention of scholars (Figure 6B). A total of 102 keywords were extracted by keyword burst analysis, the top 25 of which are shown in Supplementary Figure S3. We found that “autophagy” and “diabetic kidney disease” had the highest burst strength. In addition, we also found that “inflammation,” “protect,”, and “microRNA” were the latest keywords that emerged in the last 2 years (Figure 6C).

**TABLE 7:** Keyword cluster analysis of podocyte injury research.

Cluster ID	Size	Silhouette	Mean year	Top terms	Log (likelihood ratio)
#0	66	0.551	2009	Nephrotic syndrome	128.11
#1	60	0.735	2003	Proteinuria	76.83
#2	59	0.623	2011	Diabetic nephropathy	69.66
#3	51	0.694	2009	Oxidative stress	53.16
#4	34	0.751	2011	Autophagy	50.62
#5	31	0.61	2012	Growth	16.2
#6	31	0.734	2012	Protein	47.27
#7	22	0.779	2011	Disease	56.81

In addition, we followed the practice of (Kumar and Goel, 2021; Kumar et al., 2021) to explore the research gaps in the field of podocyte injury. We found that studies on podocyte injury were less exposed in acute kidney injury and IgA nephropathy, which may be related to the pathophysiological characteristics of podocytes. Notably, our study also found that the protective effect of traditional Chinese medicine monomer on podocyte injury is a promising blank field, and such as astragaloside iv (occurrences: 12), triptolide (occurrences: 10), berberine (occurrences: 7), etc. (Figure 6D). Xing et al. (2021) found that astragaloside iv can inhibit oxidative stress and alleviate podocyte damage. Coincidentally, Wan et al. (2021) found that triptolide can reverse the epithelial-mesenchymal transition of podocytes and improve podocyte-associated glomerular diseases. Therefore, the protective effect of traditional Chinese medicine monomer on podocyte injury needs more research to fill this gap.

## Discussion

**FIGURE 7:**
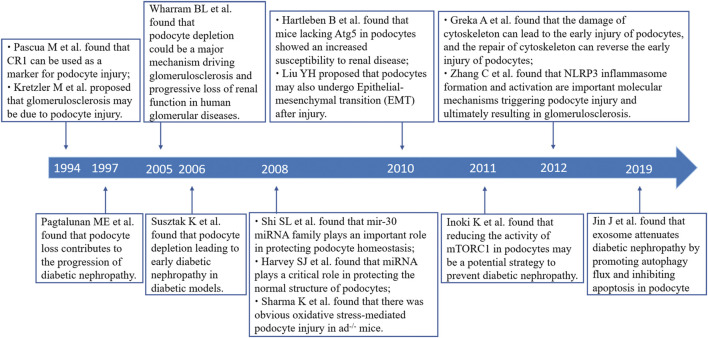
Timeline of part of landmark achievements in podocyte injury research.

In this study, we analyzed the main knowledge domain and emerging trends of podocyte injury using bibliometric analysis. Some landmark articles were also identified using this analysis (Figure 7). The results showed that the annual publications on podocyte injury generally show an upward trend and have entered a relatively mature research stage. The study of podocyte injury rose in 1994. Subsequently, Wharram et al. (2005) demonstrated that podocyte depletion may drive glomerulosclerosis and renal function progression in humans. The latest studies found that weight control was effective in preventing the kidney disease progression that occurs because podocytes fail to match the growth of the glomerular tuft (Fukuda et al., 2012).

China was the most productive country, and six of the top 10 productivity institutions are from China. Southern Medical University (published 68 articles, cited 1951 times) is the main representative, and Liu Youhua’s team from this institution contributed most of the publications, and with a long-term research focus on Wnt/β-catenin signaling and podocyte injury (Dai et al., 2009; He et al., 2011; Zhou et al., 2013). Their latest study found that the activation of the Wnt/β-catenin signaling pathway mediates oxidative stress-induced podocyte injury (Zhou L. et al., 2019). The United States was the country with eight of the top 10 most cited articles. University of Washington (published 57 articles, cited 3,986 times) is the main representative. Shankland is a leader in the field of podocyte injury at this institution and has long devoted himself to the study of podocyte injury and glomerulosclerosis. His most cited article describes the relationship between podocyte injury and glomerulosclerosis (cited 589 times) (Shankland, 2006). His latest research found that Krüppel-like factor (KLF) was closely related to podocyte injury in glomerulonephritis. KLF15 expression contributes to the stability of cytoskeleton in podocyte (Mallipattu et al., 2017), whereas the loss of KLF4 activates the STAT3 signaling pathway, which further contributes to podocyte injury (Estrada et al., 2018).

Notably, among the top 10 core journals, JASN and KI had by far, the highest in both the number of articles published and the number of citations, and indicating that these two journals are the most popular journals for scholars who study podocyte injury. Scholars all over the world hope to publish landmark articles in JASN and KI. These two journals have published the most cutting-edge results and major breakthroughs in podocyte injury research. In recent years, Both JASN and KI have mainly focused on research exploring ways of protecting podocytes from damage. For example, a recent article published in KI found that interleukin 9 plays a protective role in the context of podocyte injury (Xiong et al., 2020). In the same period, an article published in JASN found that synaptopodin conferred a protective effect against podocyte injury. Differently, JASN focuses mainly on cutting-edge research, and whereas KI pays concentrates on breakthroughs in basic research.

The timeline view of references and keywords showed that studies related to podocyte injury are mostly basic studies, such as cytoskeleton, apoptosis, autophagy, repair, and interstitial fibrosis. An important review (cited 301 times) discusses the close relationship between cytoskeleton dysfunction and podocyte injury. Cytoskeleton damage can lead to the early injury of podocytes, and cytoskeleton repair can reverse early injury to podocytes (Greka and Mundel, 2012). Similarly, the study conducted by Faul C et al. found that cyclosporine A can prevent synaptopodin dephosphorylation and promote the stabilization of the podocyte cytoskeleton to reduce proteinuria (Faul et al., 2008). Coincidentally, Liu M et al. recently found that sirt6 can maintain cytoskeletal stability and reduce podocyte apoptosis (Liu et al., 2017). Podocyte apoptosis, which is a special form of podocyte injury, exists in the early stages of DKD (Susztak et al., 2006). Schiffer M et al. found that podocyte apoptosis induced by transforming growth factor-beta existed in the early stage in FSGS mice (Schiffer et al., 2004). Oxidative stress has long been considered an important initiating factor of podocyte injury. In a highly cited article, Sharma K et al. found an obvious oxidative stress-mediated podocyte injury in adiponectin knockout (adiponectin^-/-^) mice (cited 464 times). Mo HY et al. found in their latest study that C-X-C chemokine receptor type 4 plays an important role in podocyte injury mediated by oxidative stress and can be used as a new target to improve podocyte injury (Mo et al., 2017).

Among the top 15 most cited articles, studies on DKD accounted for 46.67% (7/15), indicating that DKD is a hot focus of podocyte injury research. Pagtalunan ME et al. first found that podocyte loss was associated with DKD progression in Pima Indians with type II diabetes Pagtalunan et al. (1997). Subsequently, Nakamura T et al. found that the detection of podocytes in the urine was associated with the disease activity of DKD (Nakamura et al., 2000). At the same time, Coimbra TM et al. found that podocyte injury precedes glomerulosclerosis in diabetes mellitus (Coimbra et al., 2000) and that high glucose- and hypertension-related mechanical stress causes podocyte injury and proteinuria, which further progress to early DKD (Wolf et al., 2005). Isermann B et al. found that hyperglycemia caused the interruption of crosstalk between vascular compartment and podocytes, resulting in DKD (Isermann et al., 2007). Identifying new mediators of crosstalk between the vascular compartment and podocytes helps identify more effective treatments for DKD (Siddiqi and Advani, 2013).

With the development of podocyte damage research, some emerging research fields are gradually becoming the topics of interest of researchers. References and key bursts showed some items has had the highest bursts strength in the past 3 years. One of which is autophagy. Substantial evidence indicates that podocyte autophagy is a protective mechanism in kidney disease (Zeng et al., 2014). A landmark article on podocyte autophagy was published in 2010 by Hartleben B et al. who found that mice lacking autophagy-related 5 in podocytes showed an increased susceptibility to renal disease. Thus, they proposed that autophagy is a protective mechanism against glomerular injury (Hartleben et al., 2010). Lenoir O et al. found that hyperglycemia led to enhanced autophagic flux under the diabetic milieu, wherein autophagy in endothelial cells and podocytes inhibit the progression of DKD to glomerulosclerosis (Lenoir et al., 2015). A growing number of protein molecules that protect podocytes from injury by modulating autophagy have been found gradually, include Sirt6 (Liu et al., 2017), heme oxygenase-1 (Dong et al., 2015), and hepatocyte growth factor (Hou B. et al., 2020). Mitophagy has recently emerged as a new focus in podocyte injury research (Li et al., 2017; Zhou D. et al., 2019). Although an increasing number of studies on autophagy have been carried out, more in-depth research on autophagy are expected to be conducted in this field in the future. Another keyword with a significant citation burst is miRNA. Research on miRNA and podocyte injury has exploded because of the critical roles of miRNA. Many miRNA have protective effects on podocytes (Shi et al., 2008; Ishii et al., 2020), such as miRNA-21 (Kölling et al., 2017), miRNA-29 (Lin et al., 2014), and miRNA-30 (Wu et al., 2014). Strikingly, exosomal miRNA seems to be the specific focus in podocyte injury (Jin et al., 2019). Inflammation is the third hotspot in podocyte injury research. Inflammation is associated with podocyte dysfunction (Reidy et al., 2014). Many scholars have found that inflammasome activation leads to podocyte injury and glomerulosclerosis (Zhang et al., 2012; Abais et al., 2013; Shahzad et al., 2015). Notably, inflammation is an important bridge to podocyte injury studies. It often mediates podocyte injury together with autophagy and oxidative stress (Hou Y. et al., 2020; Dai et al., 2021).

Our research still has some limitations. First, this study only focused on podocyte injury, and we enriched the search strategy as much as possible. Still, some studies about podocytes were not included in the analysis. Second, most of the results of this study are based on machine algorithm, which is slightly insufficient in artificial induction. Finally, some new research fields related to podocyte injury may not have been included, and which may be due to the sensitivity of machine algorithms.

## Conclusion

Using bibliometric analysis, we found that the research on podocyte injury has a good research prospect. Publications related to podocyte injury are increasing exponentially. DKD is the current focus of research in this field, and autophagy, microRNA, inflammasome, oxidative stress, and exosome are potential hotspots in podocyte injury research, which needs more focus. Notably, the protective effect of traditional Chinese medicine monomer on podocyte injury is less exposed, and more research is needed to fill this research gap.

## Data Availability

All datasets presented in this study can be found in the article and Supplementary Material.
