# Supplementary material for: Knowledge Domain and Emerging Trends in Podocyte Injury Research From 1994 to 2021: A Bibliometric and Visualized Analysis
Source: Front Pharmacol. 2021 Dec 3;12:772386. doi: 10.3389/fphar.2021.772386 (PMC8678497; doi:10.3389/fphar.2021.772386)
Supplement: Supplementary file 1 [file DataSheet1.docx]

| Topical Subject | Number |
| --- | --- |
| Podocyte injury | 1808 |
| Podocyte damage | 394 |
| podocyte apoptosis | 457 |
| Podocytopathy | 226 |
| Podocyte dysfunction | 195 |
| Podocytopathies | 120 |
| podocyte pyroptosis | 4 |

Table S1. Search results of different topical subjects.


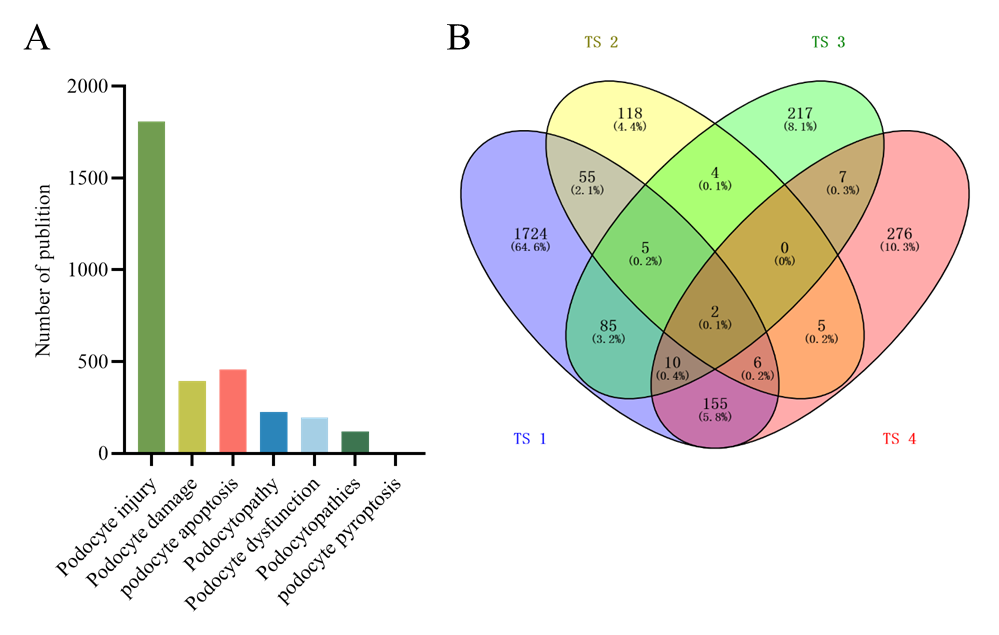


Figure S1. (A) Search results of different topical subjects; (B)Venn diagram of retrieval results of different topical subjects.

Table S2. Co-cited references cluster analysis of podocyte injury research.

| Cluster ID | Size | Silhouette | Mean year(Range) | Top term | Log(likelihood ratio) |
| --- | --- | --- | --- | --- | --- |
| #0 | 92 | 0.827 | 2011(2004-2018) | Cytoskeleton | 20.42 |
| #1 | 86 | 0.873 | 2001(1996-2007) | Diabetic nephropathy | 11.38 |
| #2 | 79 | 0.841 | 2001(1994-2011) | Stretch | 14.03 |
| #3 | 77 | 0.847 | 2012(2005-2020) | Autophagy | 59.85 |
| #4 | 74 | 0.866 | 2016(2011-2020) | Diabetic kidney disease | 20.58 |
| #5 | 68 | 0.791 | 2007(2001-2016) | Repair | 15.70 |
| #6 | 63 | 0.92 | 1996(1989-2003) | Interstitial fibrosis | 19.10 |
| #7 | 46 | 0.787 | 2008(2003-2015) | Angiotensin ii | 20.56 |
| #8 | 28 | 0.965 | 2016(2006-2019) | LncRNA | 11.89 |
| #9 | 19 | 0.901 | 2007(2003-2013) | Eplerenone | 39.28 |
| #10 | 16 | 0.991 | 2016(2012-2019) | Poria cocos | 17.69 |
| #11 | 15 | 0.978 | 2002(2001-2007) | Nephrogenesis | 8.57 |
| #12 | 11 | 1 | 1994(1991-1998) | Nuclear factor of activate T cell | / |
| #13 | 3 | 0.996 | 1995(1994-1998) | hyperhomocysteinemia | / |

Figure S2. Top 25 references with the strongest citation bursts

Figure S3. Top 25 keywords with the strongest citation bursts
